# Individual variation in spatial reference memory influences cache site choice in a wild bird

**DOI:** 10.1098/rspb.2024.3079

**Published:** 2025-03-19

**Authors:** Tas I. F. Vámos, Rachael C. Shaw

**Affiliations:** ^1^ School of Biological Sciences, Victoria University of Wellington, Wellington, New Zealand

**Keywords:** spatial memory, caching, cognition, bird, behaviour

## Abstract

The spatial cognitive abilities of food-storing birds are well documented, but how individual variation in spatial memory influences natural caching behaviour is poorly understood. Here we tested wild toutouwai (*Petroica longipes*) on two spatial memory tasks and compared their performance with caching decisions. We found that birds with better performance on a spatial reference memory task also travelled further to cache food items. As widely distributed caches are thought to offer protection against cache theft, birds with better reference memories may therefore gain greater benefits from food-storage than birds with poor memories. Females outperformed males in the spatial reference memory task, and performance also declined with age. Birds also displayed marked individual differences in how they interacted with the reference memory task, with some potentially following a heuristic to locate the reward. By contrast, birds showed no evidence that they learned the contingencies of a working memory task. Our results provide empirical evidence that individual variation in spatial memory performance influences the choices that toutouwai make during caching. We recommend that researchers seeking to link cognition and behaviour in the wild take care to select ecologically relevant cognitive tasks that are likely to underpin fitness-linked behaviours targeted by selection.

## 1. Introduction

Many animals store food for later retrieval, allowing them to survive when foraging conditions are poor or unpredictable [1]. However, the benefits of food storage are contingent on the animal’s ability to remember where cache sites are located [2]. To deal with this challenge, food-storing species are thought to possess adaptively specialized spatial memory abilities [3,4]. To date, spatial memory has primarily been studied in captivity, with a focus on the cognitive abilities involved in cache retrieval [5–9]. Consequently, there is a lack of empirical research on how individual variation in spatial memory is reflected in wild caching behaviour, such as in the number and distribution of cache sites [10,11].

The term ‘spatial memory’ has been used to describe a diverse collection of memory systems concerned with the processing, storage and use of spatial information [12]. One of these, spatial reference memory, involves the retention of spatial information that remains consistent over time [13]. Accurate reference memory appears to be essential for the recovery of caches, and food-storing birds have demonstrated the ability to use a variety of spatial cues to remember rewarded locations over long periods of time in laboratory conditions [5,14–16]. However, food-storing birds must also be able to flexibly update their memories of where stored food is located as old caches are depleted and new caches are established [17]. The rapid updating required to manage memory for cache sites shares some features of working memory, which involves the updating of short-term memories in response to new information [18]. However, caching species must retain this information for longer than the several seconds for which working memory is typically conceptualized as lasting [19]. Thus, classifying the type of memory that caching species use to remember active and depleted cache sites is challenging. However, regardless of terminology, past research suggests the ability to update short-term memory differs considerably between caching and non-caching species [20], and between caching species of varying degrees of specialization toward caching [21], even if the retention intervals involved in such studies are 5 or more minutes.

To date, most research on spatial memory abilities in food-storing species has focused on their use during retrieval, but there is evidence that they also influence the decisions made during the caching process itself [11]. Both wild Canada jays (*Perisoreus canadensis*) and captive black-capped chickadees (*Poecile atricapillus*) have been shown to preferentially create novel cache sites further away from previously made sites, with results suggesting that birds were relying on spatial memory rather than direct visual cues to do so [11,22]. The social context in which caching occurs also influences caching decisions, with individuals typically creating more scattered caches in the presence of potential conspecific pilferers [23–26]. However, the safety afforded by creating many scattered cache sites may place an increased load on the bird’s spatial memory, raising the risk of forgetting cache site locations or their contents (i.e. whether they have been retrieved yet). Therefore, an individual’s caching choices are likely to be linked to their spatial memory abilities, with reference memory influencing the distribution of sites throughout a bird’s territory and working memory potentially influencing the number of caches that they can keep active at any one time. Although a correlation between individual rates of caching and spatial reference memory performance has been found previously [27], how the decisions made during cache creation are related to individual variation in spatial memory performance is currently unknown.

Here we aimed to test whether caching decisions are linked to individual differences in spatial reference memory and spatial working memory. We examined how memory task performance influenced the caching decisions of wild North Island robins (*Petroica longipes*), henceforth referred to by their Māori name: toutouwai. The toutouwai is a food-storing passerine that caches invertebrate prey over relatively short time-scales, with retrieval typically occurring within several hours to several days of storage [28,29]. We have previously demonstrated that individual birds will adopt a consistent strategy when caching, either preferring to clump many items in a few nearby sites or to scatter caches across several distant sites [30]. Furthermore, toutouwai are easily trained to interact with experimental apparatuses, making them ideal for field-based cognition studies, including assays of individual spatial cognitive ability [31,32]. To evaluate the relationship between spatial memory and cache creation decisions in toutouwai, we first assessed individual variation in cache site choices (the methods and data are described in detail in [30]). We measured the distance that birds travelled to cache food items, as well as the number of sites they used to cache a standardized number of food items. We then tested these same individuals on a spatial reference memory task measuring the retention of information about a single rewarded location in an array of possible locations, followed by a spatial working memory task assaying individual differences in short-term memory for recently depleted locations in an array. We hypothesized that individuals with superior spatial reference memory abilities would use more sites and choose sites that were further from the food source, as their memory abilities should allow them to engage in a scatter-hoarding strategy with less risk of forgetting. Meanwhile, we expected spatial working memory performance to correlate with the number of sites used, because better working memory may allow an individual to update their content memories for more sites during cache creation and retrieval. Finally, we predicted that spatial working memory would not influence caching distance, as we expected accurate memory for widely scattered sites to be under the domain of reference memory alone.

## 2. Methods

### (a) Study site and subjects

The subjects were wild toutouwai residing in Zealandia Ecosanctuary in Wellington, New Zealand. This study site and population have been described in detail elsewhere [31]. We first ran caching sessions from April to June 2021 on 44 birds (*N*_males_ = 23, *N*_females_ = 21); these data and methods have been previously published and are described in detail in Vámos & Shaw [30]; we also describe them briefly below. We administered the spatial reference memory task from June to August 2021 using 38 (*N*_males_ = 21, *N*_females_ = 17) of the birds that had completed these caching sessions. Of these birds, we also tested 23 (*N*_males_ = 12, *N*_females_ = 11) on the spatial working memory task. All birds were tested in the same order: caching sessions first, reference memory task second and working memory task last. The working memory task was delayed due to the effects of COVID-19 lockdowns in New Zealand; due to time constraints, we had to conduct it in April–May 2022 with a smaller sample size than we had originally planned.

### (b) Caching sessions

**Figure 1.**
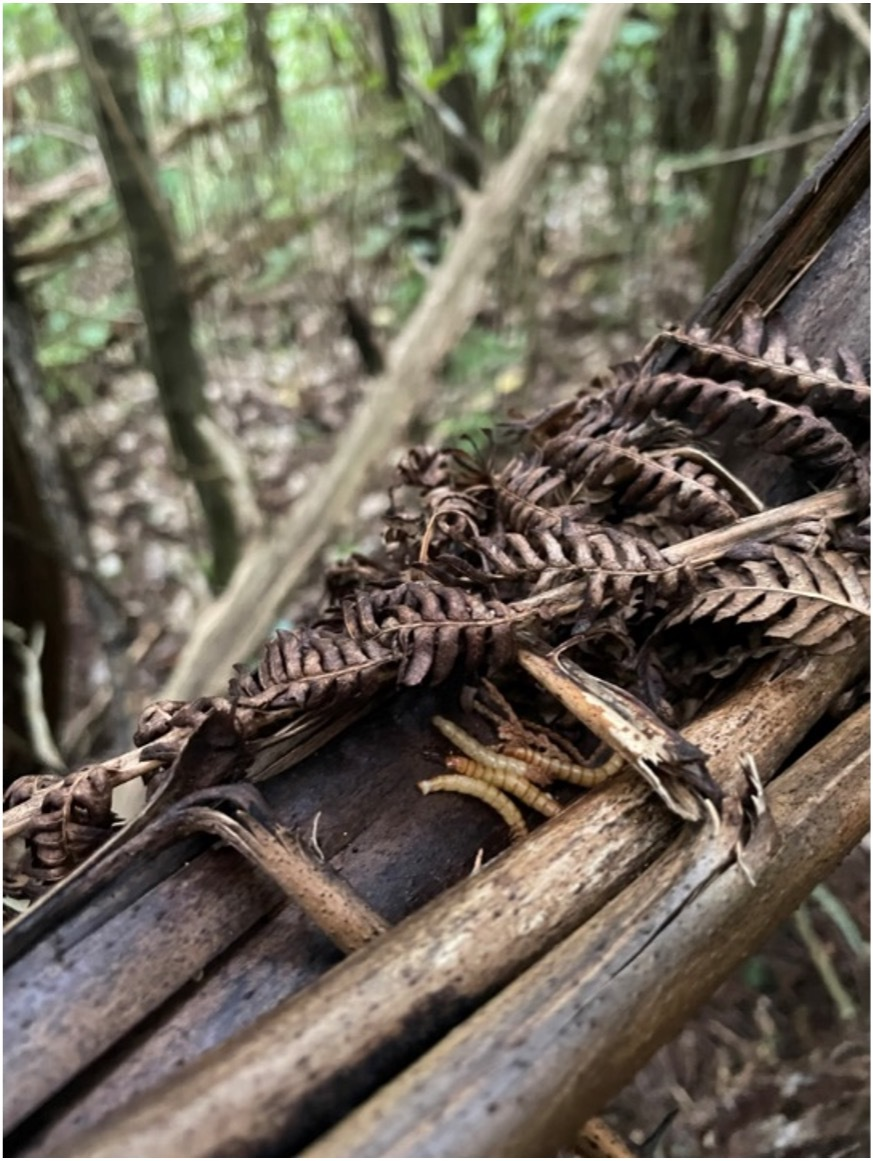
A toutouwai cache site on the stem of a desiccated fern frond, containing four cached mealworms. Stored food items are typically placed in a sheltered location such as a small cavity or groove, but no further action is taken to conceal them.

We administered three caching sessions to each bird at the same time of day on consecutive days. If inclement weather prevented data collection, the next session occurred on the next possible day. In each session, we provided the bird with one freshly killed mealworm (*Tenebrio molitor* larvae) at a time by placing it on a small wooden platform in a central location in their territory. If the bird ate the mealworm, we immediately replaced it with another. If the bird cached the mealworm, we noted the storage location before providing the next mealworm. This continued until the bird had cached five items. In rare cases where a bird’s precise cache site could not be determined (40 instances, or 4.9% of the total number of mealworms cached), we continued providing mealworms until five known caches were made. We measured the straight-line distance in metres from the feeding location to each cache site using a tape measure for sites less than 5 m away, and a Bushnell range finder for sites more than 5 m away. We recorded the number of mealworms cached in each site and photographed each site (figure 1). We tested each bird with no other conspecifics present. We used an iPhone to video record sessions. The full protocol for the caching sessions, as well as the repeatability of caching measures and relationships between measures are described by Vámos & Shaw [30].

### (c) Spatial memory tasks

**Figure 2.**
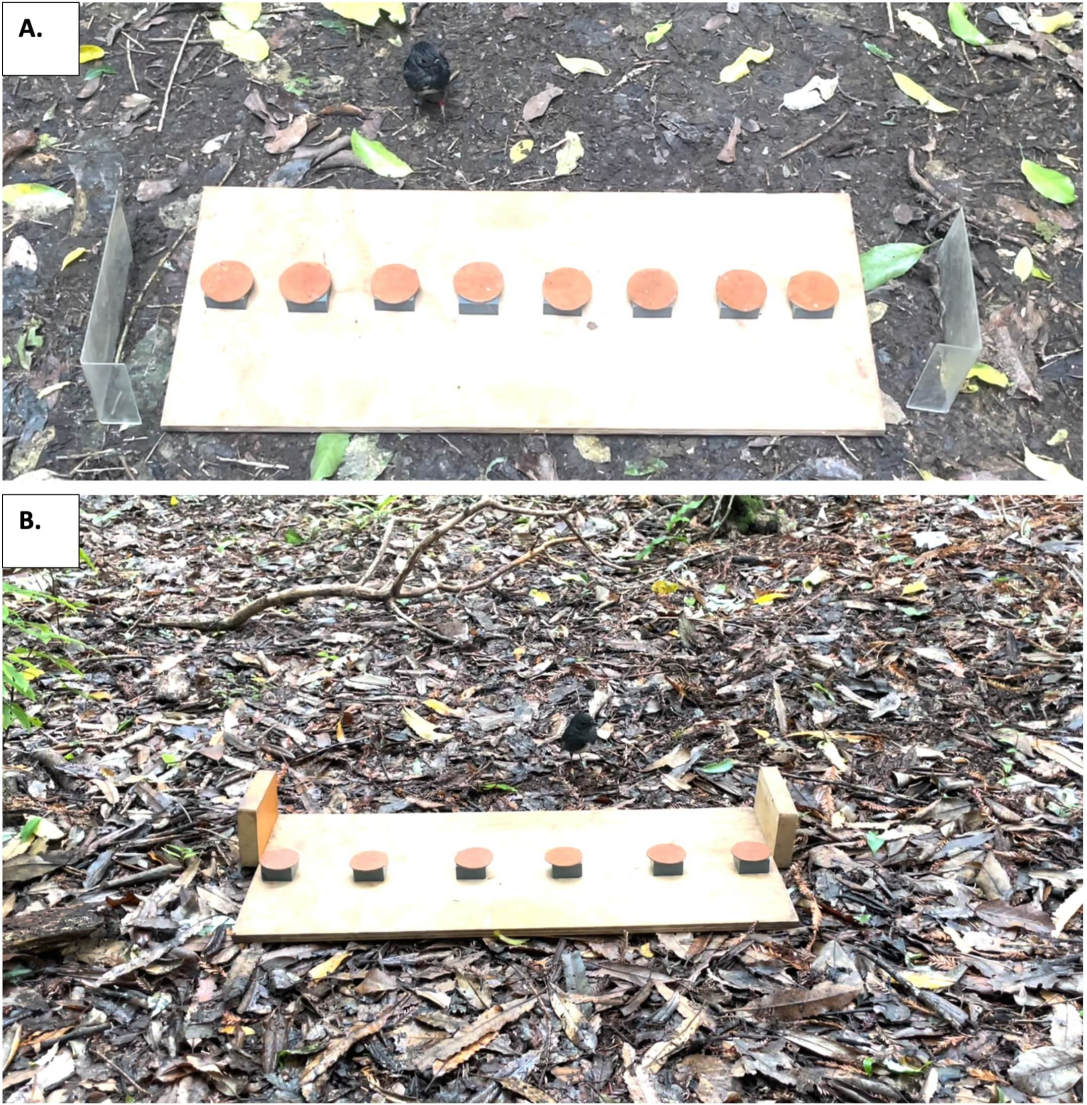
Apparatus set-up for the spatial reference memory task (A) and spatial working memory task (B). Photographs are taken from the experimenter’s position behind the board, with the bird approaching the tile array from the opposite side before deciding which lid to flip.

We trained birds to flip circular brown leather lids (5 cm diameter) covering 1 cm deep, 2 cm diameter wells drilled into square, grey plastic tiles (4 × 4 cm), in which mealworm rewards could be concealed. The training protocol for lid-flipping is described by McCallum & Shaw [33]. In both tasks, the tiles were arrayed on a wooden board (85 × 30 cm) (figure 2). General testing procedures were the same across both tasks. On testing days, we set up the apparatus in a preselected testing location in the focal bird’s territory. We selected testing locations that were not directly adjacent to any large feature that might be used as a landmark and that had no overhanging vegetation less than 1 m above. For both tasks, the experimenter (T.I.F.V.) was seated approximately 1 m behind the board, while the bird was allowed to approach from the opposite side. Both tasks used a linear array of tiles, as pilot experiments revealed that when faced with an array with multiple rows, toutouwai could not inhibit flipping lids in the first row they encountered. To reduce directional bias in the bird’s approach, we always placed two barriers (21 × 10 cm) perpendicular to the ends of the linear array, preventing the birds from approaching from one end.

Both tasks took place on consecutive testing days and included a 2 min time-out rule—if a bird did not interact with the apparatus during this time, we reset the trial. Three consecutive time-outs resulted in the task’s suspension until the next testing day; this only occurred twice, and in both cases, the bird eventually completed the task. Testing days were always consecutive, unless inclement weather prevented experimentation, in which case we resumed the task on the next possible day. After the final trial of each testing day, we weighed the bird by letting it retrieve a mealworm from an electronic scale, with the weight divided by the bird’s tarsus length as a measure of body condition [31].

#### (i) Spatial reference memory task

The spatial reference memory task measured an individual’s ability to remember a single rewarded location out of eight possible locations over the course of 30 trials, given across 2 days. The experimental set-up consisted of eight tiles with leather lids spaced 5 cm from each other in a linear array on the board (figure 2A). During testing, the third tile from the end of the array was rewarded for all birds and in all trials; we randomly selected the side of the array that contained the rewarded tile for each bird by tossing a coin. In each trial, we allowed the bird to approach the array and flip as many lids as required to retrieve the concealed mealworm. During the first trial only, the bird was also required to flip any remaining lids until all tiles were uncovered. This initial probe trial ensured the bird experienced that only one tile was rewarded. After the bird flipped the final lid, the trial ended; all subjects flipped all lids on this preliminary trial. During the intertrial interval, we covered the apparatus with a towel for 30 s to occlude the bird’s vision while we replaced all lids and placed a new mealworm in the rewarded tile. We also randomly shuffled the tiles on the board to prevent the bird from using any visual imperfections of individual tiles as a cue. After the intertrial interval, we uncovered the board and allowed the bird to approach and flip as many lids as required to find the mealworm (electronic supplementary material, video S1). In all trials after the first, we ended the trial immediately after the mealworm was retrieved, regardless of how many other lids were left unflipped. We gave each bird two sessions at the same time of day on consecutive testing days. The bird received 15 trials including the initial probe trial during its first session, and 16 trials on its second session, with the final trial being unrewarded to ensure that birds were not using olfactory cues to locate the rewarded tile. The individual performance measure in this task was the total number of lid flips that a bird made across all trials except the first (as spatial memory could not be used in this probe trial), with a lower score indicating better spatial memory through fewer errors.

#### (ii) Spatial working memory task

The spatial working memory task measured the ability of birds to avoid previously rewarded spatial locations, using an analogue of the classic radial arm maze [34]. The experimental set-up consisted of six tiles with leather lids arrayed in a line, with tiles spaced 8 cm from each other. However, to provide visual differentiation from the previous task and to further prevent the bird from approaching at an angle, we placed wooden barriers on either side of the board instead of the clear plastic barriers of the previous task (figure 2B).

During testing, we rewarded each of the six positions with a single mealworm. Except for the first day, we gave each bird a single test trial per testing day. On the first day of testing, we also gave each bird a single preliminary training trial: the bird was allowed to flip all lids and retrieve all mealworms without interruption, to inform it that all positions were rewarded. All birds successfully completed this initial trial. Immediately after this training trial, we gave the bird its first test trial. For each test trial, we rewarded all positions and allowed the bird to approach and flip one lid to retrieve a hidden mealworm (electronic supplementary material, video S2). We then approached the array, interrupting the bird before it could flip another lid. We replaced the lid on the now-empty tile and remained at the apparatus for a 30 s interval before retreating to the observation position, allowing the bird to approach once more. Unlike the previous task, the apparatus was not covered during intervals and always remained visible to the bird so that it was clear that the tile was not being rebaited. A bird that remembered previously depleted locations was expected to learn to avoid previously flipped lids. After six lid flips in this manner, we ended the trial and removed all lids, allowing the bird to retrieve any remaining mealworms. We instituted a six-flip limit because accuracy typically declined considerably as the trial progressed, resulting in many unrewarded visits, causing the birds to lose motivation to participate and refuse to flip further lids. The free retrieval gave birds the opportunity to learn that only tiles that remained unvisited at the end of the trial were still rewarded. We gave each bird one test session (and therefore one trial) at the same time each day for 15 days; the 24 h gap between trials aimed to reduce interference from birds’ knowledge of reward locations in previous trials. The individual performance measure in this task was the summed number of novel positions searched across all 15 trials, with a higher score indicating that a bird was better able to avoid revisiting depleted positions, thus indicating better spatial working memory.

### (d) Analyses

All analyses were carried out in R v. 4.3.2 [35], using the packages lme4 for mixed models [36] and rptR for repeatability estimation [37]. We first established whether the group-level performance in each of our memory tasks showed evidence that birds were in fact relying on memory to solve the task. To determine whether the birds showed evidence of learning the rewarded position in the spatial reference memory task, we used a Poisson generalized linear mixed model (GLMM) with the number of lids flipped in each trial as the response variable, trial number and the session body condition score as fixed factors and individual bird as a random factor. The session body condition score was included in this model to assess the possibility that task performance was confounded by changes in individual’s hunger/motivation across the two sessions. To investigate whether birds could have used olfactory cues rather than spatial cues to locate the rewarded position, we compared the number of errors made in the unrewarded control trial (trial 31) with the errors made in the final rewarded trial (trial 30) using a Wilcoxon signed-rank test. We used a Wilcoxon one-sample signed-rank test to evaluate whether birds were performing better than the random search expectation in their final test trial, with random search performance defined as 4.5 flips to acquire the reward (following [38]). To investigate the possibility that birds were using a heuristic to locate the reward (e.g. flipping their first lid at the end of the array closest to the rewarded position and then sequentially moving inwards towards the reward), we recorded whether the bird’s first searched position was at the end of the array closest to the reward (a binary score of 0/1 per trial). We calculated adjusted repeatability for this behavioural measure using a binomial mixed-effects model, with individual identity as random factor and trial number and body condition as fixed factors [39]. To determine whether this apparent heuristic strategy was related to overall performance on the task, we used a Poisson GLM to test whether the total number of trials in which the bird began at the end of the array nearest to the reward (hereafter called the heuristic score) was influenced by their reference memory performance score, with sex and cohort included as additional variables in the model.

Working memory is crucial for holding and updating information over the short term [18]. Therefore, rather than predicting that performance in the working memory task should improve over time, we instead examined whether this task captured consistent individual variation in working memory performance. For this, we calculated the adjusted repeatability in the number of novel positions that a bird searched in each trial, controlling for the day-to-day fluctuation in body condition and trial order, as birds might improve over time through learning general task contingencies. Repeatability was quantified using mixed-effects models [39], with the number of novel positions searched during a trial as the response variable, individual bird as a random effect, and body condition and trial number as fixed factors. In the working memory task we also examined whether birds performed better as a group than a random search expectation, suggesting that they were relying on memory. For this analysis, we used a one-sample Wilcoxon signed-rank test to compare birds’ actual performance, measured as the total number of novel positions searched in all trials, against the expected random search performance. We calculated the random search expectation as a total of 60 novel locations searched across all 15 trials (as the random search expectation for a single trial is 3.99 novel locations searched to find the reward, following Spetch & Wilkie [40]).

For the overall performance measures in both the spatial reference memory task and the spatial working memory task, we examined the influence of sex and cohort. Cohort was defined as the start of the breeding season in which the bird hatched (e.g. a bird hatched in the austral summer of 2020—2021 was assigned to cohort 2020). Immigrants to the population that were banded as adults were assigned to the previous year’s cohort. For each spatial memory task, we used a Poisson generalized linear model (GLM) with the task performance measure (summed error number across all trials for reference memory and summed novel positions searched across all trials for working memory) as the response variable, with sex and cohort as predictors. Additionally, we examined whether there was a correlation between our two spatial memory task measures using a Spearman’s rank test.

Our analysis of the working memory task performance revealed that birds did not learn the contingencies of the working memory task (see §3). We therefore excluded working memory performance from our analyses comparing spatial memory performance with caching measures, conducting these with reference memory performance only. To examine the influence of spatial reference memory performance on caching decisions, we selected two caching measures that we predicted could be influenced by spatial reference memory: (i) the straight-line distance (m) between the feeding site and cache site in each caching trial (*N*_trials_ = 15), and (ii) the total number of unique sites used across all caching trials. To investigate the influence of spatial reference memory performance on caching distance, we used a linear mixed model (LMM) with distance (m) as the response and individual identity as a random factor. Sex and ontogeny have previously been shown to influence caching behaviour in toutouwai [28,30,41], and so we also included sex and cohort as fixed factors in the model. Finally, as our heuristic score was unrelated to reference memory performance, we included it as an additional fixed factor to explore whether birds that were more likely to use heuristics may have been more prone to using the same cache sites. We predicted that birds with better spatial reference memory task performance would be more likely to cache food items further from the location of food acquisition. We used a Poisson GLM to investigate how spatial reference memory performance influenced the total number of unique sites a bird used across all 15 caching trials. We predicted that birds with better spatial reference memory should use more sites. Our previous research suggests that in non-social conditions, caching commences after birds reach satiation and two proxies for satiation (number of mealworms eaten during a caching session and time until retrieval) are not correlated with any of the spatial caching measures used here [30]. Thus, we did not include any measures of satiation in our models.

## 3. Results

Birds (*n* = 44) travelled a mean ± s.e. distance of 5.24 ± 0.09 m (range = 1.7–12 m) to cache each mealworm and created a mean of 4.47 ± 0.33 unique sites (range 1−10) across their 15 caching trials. Our previous work has shown that the distance travelled to cache and the number of sites used during these caching trials were repeatable and positively correlated with one another [30].

**Figure 3.**
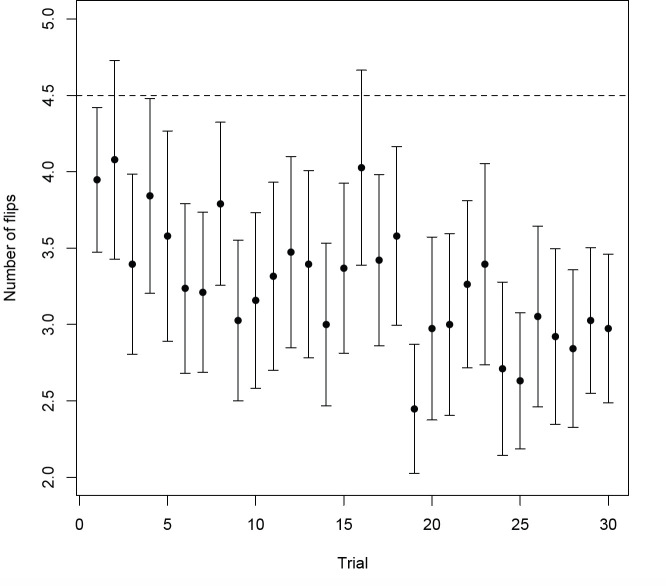
Mean number of lid flips per trial across the 30 test trials of the reference memory task. Error bars represent 95% CIs. The dashed line indicates predicted chance performance of 4.5 lids flipped.

In the spatial reference memory task, birds (*n* = 38) committed a mean of 98 ± 2 lid flips across the 30 trials after the initial probe. Birds showed evidence of learning by making fewer errors in later trials (figure 3; GLMM, trial coefficient estimate [CE] = −0.08, 95% confidence interval [CI] = −0.115 to −0.046), with no effect of body condition on task performance (GLMM body condition CE = 0.005, 95% CI = −0.516 to 0.382). Performance on the final rewarded trial (test trial 29) did not differ from performance on the control trial (test trial 30) (Wilcoxon sign rank test, *V* = 242.5, *p* = 0.842), indicating that birds were not relying on olfactory cues to retrieve the reward. As a group, by the final test trial, birds were also finding the reward in significantly fewer flips than would be expected if they were searching randomly (Wilcoxon one-sample signed-rank test: *V* = 70.5, *p* < 0.001). There was a significant relationship between the spatial reference memory performance measure and both sex and cohort: females made fewer errors in total than males, and younger birds made fewer errors in total than older birds (GLM, sex CE = 0.078, 95% CI = 0.012 to 0.144; cohort CE = −0.017, 95% CI = −0.028 to −0.005). Individual birds exhibited low but significant adjusted repeatability in their propensity to begin trials by first flipping the lid at the end of the array that was closest to the reward (*R* = 0.06, *p* < 0.001), which did not depend on trial number or body condition (GLMM, trial CE = 0.012, 95% CI = −0.007 to 0.031; body condition CE = 1.898, 95% CI = −0.170 to 8.882). Finally, males were more likely to start searching from the edge of the array than females (GLM, sex CE = 0.301, 95% CI = 0.009 to 0.597), while there was no effect of cohort (GLM, cohort CE = −0.008, 95% CI = −0.058 to 0.044), or reference memory performance score (GLM, reference memory performance CE = −0.003, 95% CI = −0.015 to 0.009). Example histograms showing the distribution of first flips can be found in electronic supplementary material, figure S1.

**Figure 4.**
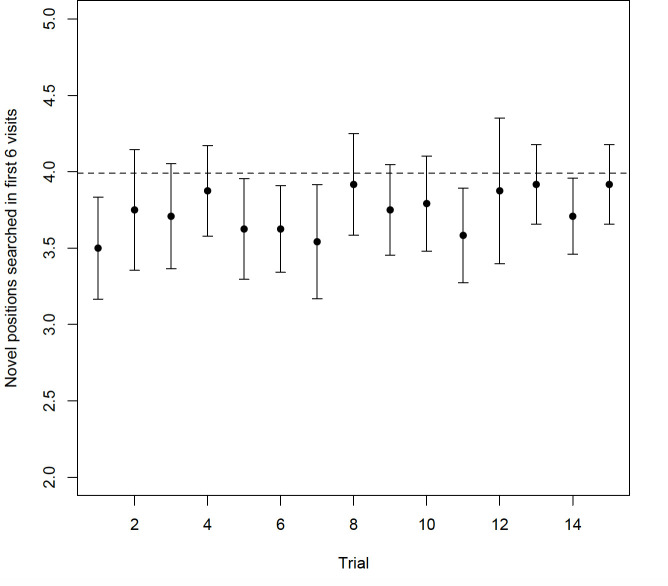
Mean number of novel positions searched during the first 6 visits to the array across the 15 trials of the spatial working memory task. Error bars represent 95% CIs. The dashed line indicates predicted chance performance of 3.99 novel positions searched.

In the spatial working memory task, birds (*n* = 23) searched a mean ± s.e. of 56 ± 1 novel positions across all 15 trials (figure 4). There was no repeatability in performance (adjusted *R* < 0.001, *p* > 0.99) when controlling for the potential confounds of trial number and body condition. The performance measure of total number of novel positions searched across all trials was not related to sex or cohort (GLM, sex CE = −0.057, 95% CI = −0.168 to 0.054; cohort CE = −0.007, 95% CI = −0.028 to 0.013). In fact, across all trials, birds performed significantly worse than the random search expectation (Wilcoxon sign rank test, *V* = 18, *p* < 0.001). Finally, there was no relationship between spatial working memory performance and spatial reference memory performance (Spearman’s rank test, *S* = 2005.8, *p* = 0.968).

**Figure 5.**
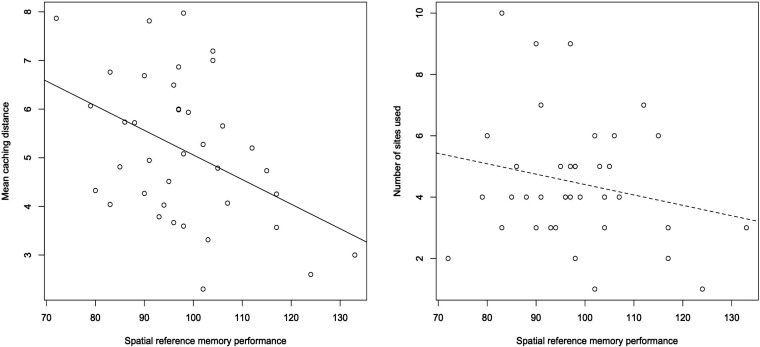
The association between spatial reference memory performance and caching measures. The left panel shows the relationship between spatial reference memory and caching distance (m). The right panel shows the relationship between spatial reference memory and site number. The lines represent the linear regression between the two variables of each comparison, with a solid line indicating a significant relationship between the performance measure and caching measure, and a dashed line indicating no significant relationship.

As birds showed no evidence of using spatial working memory, we did not examine the relationships between our measures of caching behaviour and the spatial working memory task performance. However, there was an association between spatial reference memory performance and the total distance travelled during caching sessions; birds that made fewer reference memory errors cached items further away (table 1; figure 5). There was no such relationship between spatial reference memory performance and the number of cache sites used (table 1; figure 5). There was no effect of sex, cohort or heuristic use on either measure of caching (table 1).

**Table 1.** Model results for spatial memory-caching measure comparisons. GLM, generalized linear model; LMM, linear mixed model. Bold type indicates significance as 95% confidence intervals that do not include 0.

model	response	term	estimate	s.e.	95% CIs
LMM *n* = 38	caching distance (m)	intercept	243.035	172.933	−81.298 to 567.371
		**spatial reference memory**	−**0.050**	**0.020**	−**0.088 to −0.012**
		sex	−0.819	0.477	−1.714 to 0.077
		cohort	−0.115	0.085	−0.275 to 0.045
		heuristic score	0.034	0.064	−0.086 to 0.154
Poisson GLM *n* = 38	no. of cache sites used	intercept	35.963	61.368	−86.842 to 153.935
		spatial reference memory	−0.007	0.007	−0.022 to 0.007
		sex	−0.190	0.169	−0.520 to 0.142
		cohort	−0.017	0.030	−0.075 to 0.044
		heuristic score	0.033	0.023	−0.012 to 0.077

## 4. Discussion

Spatial memory is an essential cognitive ability for food-storing animals. For the first time, we show that individual differences in spatial memory performance influence cache placement decisions in the wild. We examined individual performance on two tasks testing different kinds of spatial memory—reference memory and working memory. While we found no evidence that birds were using working memory in our task, we did find a clear learning effect in the spatial reference memory task, suggesting that it did capture reference memory performance. We also found evidence that some birds may have employed a heuristic strategy in which they began each trial by flipping the first lid at the end of the array closest to the reward and then flipped each adjacent lid sequentially, moving inwards along the array until reaching the reward. Finally, comparing spatial reference memory performance to caching behaviour, we found that memory performance was associated with the distance that birds chose to carry food away from the source before caching, with birds with more accurate spatial reference memory performance caching items further away, and worse performing birds caching items closer to where they were acquired.

Given the association that we found between spatial reference memory performance and caching distance, our results raise the possibility that birds with superior spatial reference memories could benefit from a reduced risk of cache theft by being able to scatter caches more widely [26]. If this is the case, birds with poorer spatial reference memory performance may clump cache sites closer together out of necessity, as they otherwise risk forgetting the locations of spread-out cache sites. This interpretation assumes that the choices that birds make during caching reflect inherent limitations imposed by their individual spatial reference memory performance. This would indicate a unidirectional relationship between spatial memory and caching, and we should expect variation in both traits to be heritable [42,43]. Caching behaviour—including caching distance and site number—is repeatable across years in toutouwai, which may indicate heritability [30]. A previous study with toutouwai found that better reference memory performance (using a different apparatus, with fewer trials and longer retention intervals than we used in this study) was positively associated with the number of independent offspring produced by males [32]. While this previous finding is consistent with selection acting on spatial reference memory in toutouwai, its heritability and link to caching were not quantified in that study. Variation in spatial memory ability, such as we observed in this study, could also be maintained in the population if poorly performing birds possess traits that negate the costs of poor memory, such as increased boldness/aggression leading to more successful cache defence [44].

A body of research on several species suggests that spatial memory exhibits considerable plasticity. For instance, various food-storing birds and brown-headed cowbirds (*Molothrus ater*) show plasticity in neural regions associated with spatial memory that correlate with seasonal increases in caching and brood parasitism, respectively [45,46]. In humans, the spatial memory demands placed on London taxi drivers during navigation training have been linked to increased grey matter [47]. This raises the intriguing possibility that individual differences in toutouwai spatial memory may instead arise from variation in caching experiences. Responding to the risk of cache theft is one context in which toutouwai may experience variation in the cognitive demands associated with caching. In this scenario, birds with repeated experience with pilferers would naturally engage in more scattered caching, thereby ‘training’ themselves to have better spatial memory abilities driven by the necessity to retrieve scattered caches. Meanwhile, birds that experience less cache theft, for example by being better able to actively defend sites, or by selecting more concealed cache sites, would receive less natural ‘training’ for better spatial memory ability. The toutouwai’s sister species, the kakaruwai (South Island robin, *Petroica australis*), can modify its caching behaviour and scatter sites further away after experiencing cache theft [48], but whether there is also an effect of this experience on spatial memory is unknown. Unravelling the causal relationship between spatial memory and caching decisions in toutouwai will require more data on the heritability and/or plasticity of spatial memory performance.

The finding that sex and cohort influenced spatial reference memory performance stands in contrast to previous studies on toutouwai, which have not found sex differences in cognitive abilities, including spatial memory [31–33]. Our protocol, which involved more trials and shorter retention intervals than these previous assays of toutouwai reference memory, may have allowed us to uncover subtle sex differences missed by previous studies. However, these sex-based differences in spatial memory performance did not carry over into the caching behavioural measures we examined here (but see [30] for evidence that female caching behaviour can differ from males in some aspects). Potentially, sex differences in spatial reference memory could be expressed more strongly in aspects of caching that we did not examine in the current study, such as in observational spatial memory (e.g. females are more likely to pilfer caches [41]). Younger birds outperformed older birds on the spatial reference memory task, potentially due to age differences in neophobia and/or boldness, which have been documented in several taxa, influencing task performance [49–51]. In addition, senescence of memory in older birds could play a role in their inferior performance, as has been demonstrated in pigeons (*Columba livia*) [52]. Although previous work on food-storing mountain chickadees found no evidence of senescence of spatial memory in subjects between the ages of 1 and 6 [53], with findings suggesting that chickadees improve on task performance with age [54], our study population encompasses individuals from less than 1 to at least 12 years old. The comparatively long lifespan of toutouwai may thus allow greater opportunity for senescence to occur. However, as with sex, age had no effect when included in the caching models, suggesting that age-related differences in spatial memory did not carry over into caching.

Examining how individuals first approached the spatial reference memory task in each trial revealed that some individuals may have employed a heuristic to solve the task, beginning at the end of the array closest to the reward and then moving inwards towards the reward location. In our spatial reference memory task, this approach still allows birds to locate reward in fewer flips than would be expected by random search alone—provided the bird begins from the correct side, the reward will always be the third in the sequence of flips, as opposed to a mean of 4.5 flips if positions are searched randomly. In humans, a greater cognitive load is associated with an increased reliance on heuristics [55]. Potentially, toutouwai that exhibited the ‘edge effect’ heuristic faced more distractions during testing, e.g. from stressors such as territory maintenance. As male toutouwai exhibit greater territoriality than females [56], this could explain why males were also more likely than females to begin trials on the array’s edge. Individual variation in the use of heuristics remains a poorly understood phenomenon in animal cognition [57,58]. Nonetheless, our results indicate that individuals of the same species can employ multiple ways to solve the same spatial task. This individual variation in solving strategies needs to be carefully considered when examining the behavioural and fitness correlates of cognitive task performance.

Individual working memory performance was not repeatable across trials, suggesting that the task was not capturing individual differences in memory ability. Moreover, at the group level, birds performed worse than chance, suggesting that birds were preferentially revisiting depleted sites and thus failed to understand the task contingencies. Even though the presentation of each spatial memory task was separated by a breeding season (i.e. tasks were given to the same individuals approximately eight months apart), the working memory task was visually similar to the spatial reference memory task, which may have confounded performance. Additionally, we tested spatial working memory in the autumn, while spatial reference memory was tested in the winter and early spring. Potentially, seasonal variation in spatial cognition could have confounded our results, as brain structures associated with spatial memory are known to vary seasonally in other food-storing species [46]. This is unlikely, however, as previous research on toutouwai in our population suggests that the rate of caching remains consistent throughout the non-breeding season (approximately March–September) [59], which is the period in which we tested birds in this study. While adapting classic laboratory paradigms such as the radial arm maze to the field has been a hallmark of field cognition research [60], it is possible that different, food-storing-specific tasks need to be developed to better allow caching birds to demonstrate their adaptively specialized cognitive abilities [61]. In particular, research aimed at isolating the precise spatial cognitive mechanism that birds use to update their knowledge of active and depleted caches will allow for the creation of more precise individual assays with greater ecological relevance.

Here we provide the first empirical evidence for an association between spatial reference memory and cache placement decisions in a wild food-storing animal. Our results support the long-standing hypothesis that individual spatial memory directly influences caching behaviour, but also suggest that not all aspects of spatial memory are equally relevant to caching. More broadly, our study illustrates the importance of assessing how individual variation in cognitive ability is reflected in natural behaviours. As cognition is visible to selection via the behaviours it influences, assessing the validity of proposed cognition–behaviour relationships is an important consideration when considering how cognitive abilities such as spatial memory are subject to selection [62]. Our findings also indicate that the roles of sex and age must be considered, as well as the potential plasticity of cognition based on individual experiences. Finally, the apparent use of a heuristic by some, but not all, of our subjects highlights the potential for substantial variation in how conspecifics interact with cognitive tasks. Understanding the causes and consequences of this variation may provide insight into how variation in cognitive traits is maintained.

## Data Availability

Data and code are accessible on Dryad at https://doi.org/10.5061/dryad.sn02v6xg0 [63]. Supplementary material is available online [64].
